# Renal and Safety Outcomes of SGLT2 Inhibitors in Patients with Type 2 Diabetes: A Nationwide Observational Cohort Study

**DOI:** 10.3390/jcm14103349

**Published:** 2025-05-12

**Authors:** Junhyuk Chang, Chungsoo Kim, Heejung Choi, Rae Woong Park, Sukhyang Lee

**Affiliations:** 1 Department of Biomedical Sciences, Ajou University Graduate School of Medicine, Suwon 16499, Republic of Korea; wkd9504@ajou.ac.kr; 2 Section of Cardiovascular Medicine, Department of Internal Medicine, Yale University School of Medicine, New Haven, CT 06510, USA; chungsoo.kim@yale.edu; 3 Center for Outcomes Research and Evaluation, Yale New Haven Hospital, New Haven, CT 06510, USA; 4 Department of Nephrology, Ajou University School of Medicine, Suwon 16499, Republic of Korea; heeej.choi@gmail.com; 5 Department of Biomedical Informatics, Ajou University School of Medicine, Suwon 16499, Republic of Korea; 6 Division of Clinical Pharmacy, College of Pharmacy, Ajou University, Suwon 16499, Republic of Korea

**Keywords:** type 2 diabetes mellitus, sodium–glucose transporter 2 inhibitor, dipeptidyl peptidase IV inhibitor, kidney disease, cardiovascular disease

## Abstract

**Background/Objectives**: Evidence on the renal benefits and safety of sodium–glucose cotransporter 2 inhibitors (SGLT2i) in the Asia region is still lacking. This study aimed to evaluate the renal and safety outcomes of SGLT2is compared with dipeptidyl peptidase-4 inhibitors (DPP4i) using real-world data. **Methods**: A retrospective cohort study was conducted using the nationwide claims data in Republic of Korea. We evaluated kidney outcomes (any new-onset kidney events, acute kidney injury (AKI), chronic kidney disease (CKD), and kidney failure) as primary outcomes and safety outcomes (infection, hemodynamic adverse events, and fracture). Propensity score matching was used to adjust confounders, and the hazard ratios were calculated using the Cox proportional hazards model. **Results**: The study included 13,649 patients in the SGLT2i group and 35,043 in the DPP4i group after the matching. The SGLT2i group had a lower risk of kidney diseases, AKI, and CKD (HR 0.88 [0.61–0.74]) than the DPP4i group. For secondary outcomes, the risk of genital infection was higher (HR 2.38 [2.12–2.68]), and the risk of hyperkalemia was lower in the SGLT2i group than in the DPP4i group (HRs 0.49 [0.36–0.67]). **Conclusions**: The SGLT2 inhibitors had a lower risk of new-onset kidney outcomes and CKD than the DPP4 inhibitors. A high incidence of genital infection and a low incidence of hyperkalemia were shown in the SGLT2 inhibitor.

## 1. Introduction

The sodium–glucose cotransporter 2 inhibitor (SGLT2i), inducing glucosuria in patients with type 2 diabetes, has shown cardiovascular benefits based on the findings of several large RCTs [1,2]. Aside from its benefits for cardiovascular health, SGLT2is also showed clear benefits for renal outcomes in trials. Recently, clinical trials have been conducted to evaluate the effect of SGLT2i treatment on patients with chronic kidney disease (CKD). In the CREDENCE study, canagliflozin lowered the risk of kidney failure in patients with attenuated kidney function [3]. Regardless of the presence or absence of diabetes, lower incidences of a renal composite outcome, progression of CKD or cardiovascular death were observed [4].

It is important to investigate the consistency between the efficacy of clinical trials and the observational study using real-world data to assess generalizability in routine clinical setting. Especially, the real-world study on renal and related safety outcomes of SGLT2is are under-represented than cardiovascular outcomes. Since data from Asian populations represent only a small portion of the existing evidence, estimated at around 30%, establishing the external validity of SGLT2i outcomes using real-world data from this population remains essential. Additionally, only a limited number of studies have demonstrated that initiating SGLT2i treatment is associated with a slower decline in kidney function at the early period and improved composite kidney outcomes compared to other antidiabetic drugs in Asian populations [5]. 

Therefore, in this study, we aimed to investigate the effectiveness of SGLT2i treatment on renal outcomes and its safety compared with those of dipeptidyl peptidase-4 inhibitors (DPP4i) and to generate the real-world evidence using large claims data from South Korea.

## 2. Materials and Methods

### 2.1. Study Design and Data Sources

This is a retrospective cohort study adopting new-user active comparator design to test hypotheses evaluating the effectiveness and safety of SGLT2is compared to DPP4is [6]. We used nationwide claims data from 1 January 2018 to 30 April 2022 (4.3 years) from the Health Insurance Review and Assessment Services Common Data Model (HIRA-CDM) database in South Korea [7]. The HIRA-CDM database holds the administrative claims for the 20% sampled population (approximately 10 million) of all Korean citizens. This database includes demographics, diagnoses, medical and treatment procedures, and prescriptions on a national reimbursement list. Mortality was ascertained using national death records from Statistics Korea, which are included in the HIRA-CDM database. This database is converted into Observational Medical Outcomes Partnership common data model (OMOP-CDM) version 5.3.1. The codes for diagnosis were mapped to the Systematized Nomenclature of Medicine—Clinical Terms code (SNOWMED-CT), and the codes for drugs were mapped to the Medical Prescription Normalized code or the Anatomical Therapeutic Chemical Classification System. This study was approved by Ajou University Institutional Review Board (IRB No. 202207-HB-EX-001) and the need for written informed consent was waived under the regulations of Republic of Korea.

### 2.2. Study Population

Detailed definition of study population, code lists, and study flowchart was shown in the Supplementary File (Figures S1 and S2 and Table S1). We identified adults (aged over 30) who had a diagnosis of Type 2 diabetes mellitus (T2DM) and a prescription of metformin as first-line therapy at the time of cohort entry. The patients with T2DM were divided into two study groups: the SGLT2i and DPP4i groups. The SGLT2i group was defined as patients having at least one prescription of SGLT2i (dapagliflozin, empagliflozin, ertugliflozin, and ipragliflozin). Canagliflozin, the second SGLT2 inhibitor approved in South Korea, was not included since it voluntarily withdrew from the Korean market in June 2019 due to a lack of competitive insurance coverage. The combined products of SGLT2is and metformin were included in the list of the study drugs. The index date was defined as the date of the first SGLT2i prescription, and all patients should have at least 1 year in the enrolled period in the database before the index date. Furthermore, we excluded patients with a medication supply of less than 30 days. To investigate the effect of the SGLT2i, we also excluded patients who were prescribed both SGLT2is and DPP4is at the index date and with other antidiabetic drugs, except metformin, SGLT2is, and insulin, from before the index date to the index date. Patients with insulin were included considering the use case for a poor symptom or combined therapy in the early stage of type 2 diabetes mellitus. The DPP4i group were selected as an active comparator and defined under the same strategy as the SGLT2i group to minimize the selection bias of the disease stage of T2DM. Table S1 shows the list of the DPP4is in South Korea.

### 2.3. Clinical Outcomes and Follow-Up

We assessed the clinical outcomes in the follow-up period, which were defined based on the structured codes to identify them (Table S1). The date of occurrence was determined as the first occurrence of any code corresponding to the definition. The eGFR could not be utilized because lab values were not available in the database.

The primary outcomes were the new-onset kidney events, defined as a composite outcome that includes any types of kidney-related diseases, such as the following: acute kidney injury (AKI), CKD, and kidney failure. Each individual component was also evaluated separately. Congenital and hereditary kidney diseases were excluded in the kidney events. Due to the nature of claims data, AKI and CKD were defined based solely on diagnostic codes, as laboratory results were not available. CKD included all cases regardless of stages. However, kidney failure was defined as any records of stage 5 CKD, end-stage renal disease (ESRD), peritoneal dialysis, hemodialysis, continuous renal replacement therapy (CRRT), and kidney transplantation. Dialysis was identified based on procedures related to hemodialysis, peritoneal dialysis, and CRRT or device record (e.g., dialysis catheter) (Table S1). Kidney transplantation was defined based on both diagnosis and procedure codes. 

The secondary outcomes were safety outcomes related to the drug administration including infection, hemodynamic adverse events, and fracture. We investigated the incident cases of genital infection, urinary tract infection, diabetic ketoacidosis, hyperkalemia, hypokalemia, hypovolemia, hypoglycemia, and bone fracture. All-cause mortality and cardiovascular outcomes were also evaluated. Cardiovascular outcomes were defined as four-point MACE which included at least one of the following events: acute myocardial infarction (AMI), stroke (ischemic or intracranial hemorrhagic stroke), hospitalization with heart failure (HHF), and sudden cardiac death. In addition, AMI, stroke, and HHF were analyzed separately.

Patients were followed until the earliest date of the occurrence of study outcome, death, or last observation in the database, referred to as intention-to-treat follow-up.

### 2.4. Statistical Analysis

In the baseline characteristics, continuous variables were denoted as median and interquartile range (IQR), and the categorical variables as frequency and percentage. To address confounding bias arising from the retrospective study with nonrandomized treatment allocation, a large-scale propensity score (PS) model was applied [8]. The PS was calculated from a L1 regularized logistic regression model using over five thousands of the covariates at the baseline (i.e., age groups in 5 years, sex, index year, comorbidities within a year before the index date, concomitant drugs within a year before the index date, Charlson Comorbidity Index, Diabetes Complications Severity Index, and CHA2DS2-VASc score). The SGLT2i and DPP4i groups were matched by a greedy matching method without a replacement, with a maximum ratio of 1:4 within a caliper range of 0.2 standard deviation of PS. The standardized mean difference (SMD) was calculated to assess the balance of observed covariates between the groups before and after the PS matching. The covariates with an SMD of <0.1 was considered in balance. The incidence rate (IR) per 1000 person-years (PYs) for each outcome was calculated. The Cox proportional hazard regression models were used to estimate hazard ratios (HRs) with 95% confidence intervals (CIs) for clinical outcomes between the groups. *p*-values < 0.05 were considered statistically significant. The cumulative incidence plot is shown for visualizing incidence over time.

### 2.5. Sensitivity and Subgroup Analysis

To validate robustness of our study, extensive sensitivity analyses were conducted in varying analytic strategies: (1) follow-up period and (2) PS adjustment. First, we varied the definition of follow-up with considering the censoring events, which is also known as the as-treated follow-up. Patients were censored based on: (1) occurrence of any clinical outcomes, (2) prescription of other antidiabetic drugs as defined in the exclusion criteria, and (3) treatment discontinuation. Continuation of treatment was determined based on prescription records, which were defined as continuation if a new prescription was issued within 30 days of the previous prescription’s end date. Discontinuation was defined as having no new prescription prescribed within 30 days of the last prescription. Second, we applied additional PS matching with a 1:1 ratio, and PS stratification into five strata were applied to assess consistency.

The main population was divided into several subgroups for analysis: (1) a cardiovascular risk subgroup and (2) a renal risk subgroup. The cardiovascular risk subgroup included patients with a history of hypertension, hyperlipidemia, obesity, AMI, or stroke. The renal risk subgroup included patients with any prior renal disease such as renal impairment, nephritis, AKI, and CKD, excluding congenital or hereditary disorders.

All analyses were performed using R 3.5.1. The study package was developed based on open-source Health Analytics Data-to-Evidence Suite, maintained by the Observational Health Data Sciences and Informatics (OHDSI) initiative [9]. The study package including all analytic codes is publicly available in the online repository (https://github.com/ABMI/AntidiabeticHIRA, accessed on 22 June 2023). This study was reported in accordance with the Strengthening the Reporting of Observational Studies in Epidemiology (STROBE) reporting guidelines and joint ISPOR-ISPE guidelines [6,10].

Data for the results are available on reasonable request to the corresponding author. Inquires for original datasets should be directed to the Big Data Department of Health Insurance Review and Assessment Service (https://opendata.hira.or.kr, accessed on 26 July 2022).

## 3. Results

### 3.1. Cohort Characteristics

This analysis included 13,649 patients in the SGLT2i group and 35,043 in the DPP4i group. Table 1 presents the baseline characteristics of the main analysis before and after PS matching. After PS matching, all baseline characteristics were well balanced (all SMD < 0.10; Table 1, Figure S3). 

The median (IQR) follow-up was 597 (707) days for the SGLT2i group and 620 (698) days for the DPP4i group. Females comprised 40.1% and 40.2% of each group, respectively, with the most frequent age group being 40-59 years (50.7% and 50.8%, respectively). Hyperlipidemia (86.0% and 86.3%) and hypertensive disorder (65.1% and 65.2%) were the most common comorbidities. Lipid-modifying (52.1% and 52.2%) and antithrombotic agents (47.2% and 47.2%) were the most frequently prescribed medications.

### 3.2. Outcome Assessment

Significant differences in renal outcomes were observed between the SGLT2i and DPP4i groups (Table 2). 

**Figure 1:**
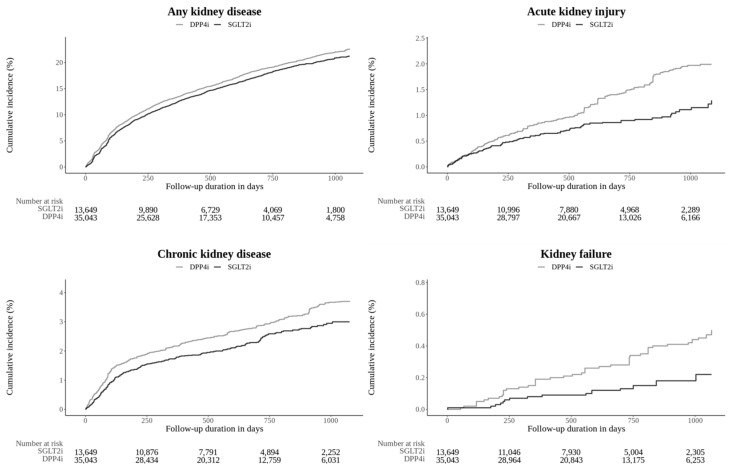
Kaplan–Meier plot of the outcomes estimated from the main analysis setting that compared the SGLT2i group versus the DPP4i group. SGLT2i: sodium–glucose cotransporter 2 inhibitor; DPP4i: dipeptidyl peptidase-4 inhibitor.

The SGLT2i group showed a significantly lower risk of the composite kidney outcome (IR: 54.75 per 1000 PYs for SGLT2i vs. 61.08 pr 1000 PYs for DPP4i; HR: 0.88 [0.81–0.96]). For individual components, the SGLT2i group showed a significantly lower risk of AKI and CKD compared to the DPP4i group (HR 0.61 [0.46–0.81] for AKI and HR 0.74 [0.60–0.91] for CKD). However, there were no significant differences in the risks of dialysis or kidney failure between the two groups. Kidney transplantation could not be assessed due to no occurrences in the SGLT2i group. The cumulative incidence plots are presented in Figure 1.

For other safety outcomes, the risk of genital infection was significantly higher in the SGLT2i group (HR 2.38 [2.12–2.68]), while the risk of hyperkalemia was lower (HR 0.49 [0.36–0.67]). The IR of diabetic ketoacidosis was low (IR 0.93 for the SGLT2i group and 0.59 for the DPP4i group) and did not show significant differences between the groups. No significant difference was found for other safety outcomes.

All-cause mortality was significantly lower in the SGLT2i group (HR 0.72 [0.54–0.93]; Table S2). All IRs for cardiovascular diseases were lower in the SGLT2i group than the DPP4i group. Statistical significance was shown for MACE (HR 0.79 [0.68–0.91]) and HHF (HR 0.75 [0.62–0.90]). 

### 3.3. Sensitivity Analysis

**Figure 2:**
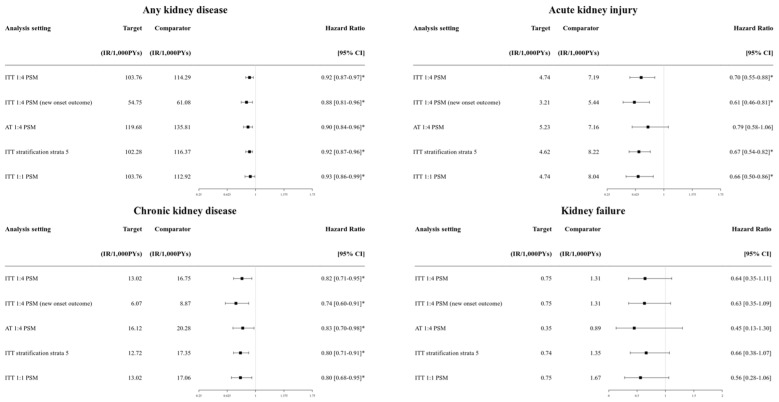
Forest plots of the outcomes estimated from sensitivity analysis setting that compared the SGLT2i group versus the DPP4i group. SGLT2i: sodium–glucose cotransporter 2 inhibitor; DPP4i: dipeptidyl peptidase-4 inhibitor. * Statistically significant.

Figure S4 shows the balances between SGTL2i and DPP4i groups in all sensitivity analyses. All comparisons in the sensitivity analyses were conducted with balanced pair groups (max SMD < 0.10). Figure 2 and Table S3 present the primary renal outcomes sensitivity analysis between the SGTL2i and DPP4i groups. 

Composite kidney outcomes and CKD were consistently lower in the SGLT2i group compared to the DPP4i group across the different follow-up strategy and PS adjustment methods (HRs ranging from 0.86 to 0.89 for any kidney outcomes; HRs ranging from 0.72 to 0.75 for CKD; all statistically significant). AKI showed consistently lower risks in the SGLT2i group compared to the DPP4i group with statistical significance, except in the as-treated follow-up (Table S3). For safety outcomes, the significance of genital infection (higher risk in the SGLT2i group) and hyperkalemia (lower risk in the SGLT2i group) were consistent across sensitivity analyses (Table S4). HHF also consistently showed a lower risk in the SGLT2i group (HR from 0.70 to 0.79; Table S4). 

### 3.4. Subgroup Analysis

In the cardiovascular risk subgroup, all baseline characteristics were balanced (all SMD < 0.10; Table S5, Figure S5). The proportions of females were 40.7% and 40.3% in the SGLT2i and DPP4i groups for patients with cardiovascular risk, respectively. Lipid-modifying agents, antithrombotic agents, and renin–angiotensin system agents were the most prescribed medications (50.3% and 54.1%, 48.2% and 48.1%, and 40.4% and 40.2%, respectively) (Table S5). In the cardiovascular risk subgroup, the SGLT2i group had a lower risk of composite kidney outcomes, AKI, and CKD compared to the DPP4i group (HR 0.90 [0.83–0.98], HR 0.53 [0.39–0.72], and HR 0.81 [0.66–0.998], respectively; Table 3). 

Other renal outcomes were not significantly different between groups. Genital infection and hyperkalemia showed significant differences (HR 2.34 [2.08–2.64], HR 0.46 [0.33–0.63]; Table S6) in line with the main analysis. Bone fracture was less frequent in the SGLT2i group with cardiovascular risk (HR 0.90 [0.82–0.98]; Table S6). The risks of MACE and HHF were lower in the SGLT2i group. In particular, the results of AKI, genital infection, hyperkalemia, MACE, and HHF were consistent across sensitivity analyses (Tables S7 and S8).

For the renal risk subgroup, all characteristics were balanced (all SMD < 0.10; Table S9, Figure S5). The proportions of females were 44.1% and 43.3% in the SGLT2i and DPP4i groups, respectively. Hyperlipidemia and hypertension were the most common comorbidities (92.0% and 92.2% for hyperlipidemia; 70.2% and 70.6% for hypertension; Table S9). AKI and CKD were significantly lower in the SGLT2i group (HR 0.40 [0.21–0.70], HR 0.68 [0.45–0.98]; Table S3). Genital infections were more frequent in the SGLT2i group, consistent with the whole study population (HR 2.41 [1.84–3.15]; Table S10). Hyperkalemia and MACE were lower in the SGLT2i group (HR 0.26 [0.14–0.46], HR 0.75 [0.56–0.98]; Table S10). However, only genital infection and hyperkalemia were consistent across all sensitivity analyses (Table S12).

## 4. Discussion

This nationwide study compared the effectiveness of SGLT2i on renal and safety outcomes to confirm its real-world benefits by utilizing routinely collected data in clinical practice. Extensive data-driven analytic methods were adopted in our study, including an active-comparator, new-user cohort study, and large-scale propensity matching. We found that SGLT2i treatment reduced the risk of composite kidney disease, AKI, and CKD. Consistent results across various analytic approaches, including sensitivity and subgroup analyses, supported the robustness of our findings. Moreover, in terms of safety outcomes, the use of SGLT2 inhibitors was associated with a reduced risk of hyperkalemia but a heightened risk of genital infections. To our knowledge, this is the largest nationwide cohort study in Asia comparing the risks of kidney and related safety outcomes between patients with SGLT2is and DPP4is.

Recently, the reno-protective effects of SGLT2is have gained as much attention as its cardiovascular benefits. The SGLT2i confers renal protection by increasing the urinary excretion of glucose and sodium, thereby lowering blood glucose levels. This mechanism of action activates tubuloglomerular feedback, leading to the afferent glomerular arteriole, which reduces intraglomerular pressure, and protects the glomeruli. Additionally, the SGLT2i initially decreased the glomerular filtration rate, thereby reducing the tubular workload and metabolic demand, while improving renal cortical oxygenation [11]. Collectively, these effects of SGLT2is extend beyond glycemic control, helping to lower the risk of AKI and slow the progression of CKD [12].

Other real-world studies have shown renal benefits of the SGLT2i across AKI, CKD, and ESRD [13,14]. In our study, however, our study did not show significant effects on dialysis initiation, kidney failure, or kidney transplantation. This may be due to the inclusion of a less severe, new-user cohort and a relatively short follow-up period, particularly in relation to the duration of T2DM. Further evaluation is needed in patients with more advanced disease, especially those with established CKD. 

Furthermore, our study confirmed consistent results for infectious and hemodynamic safety outcomes. It has already been proven in clinical trials and observational studies that SGLT2is increase the risk of genital infections due to increased urinary sugar levels. Our study also found the risk ratio of genital infection to be more than twofold. While some studies have reported an increased risk of urinary tract infection [15], this was not significant in our study, nor in other large studies [16]. However, this result may highly rely on the detailed definition of urinary tract infection and the code validity. Therefore, further research is needed to investigate urinary tract infections more thoroughly.

Hyperkalemia is a critical condition that increases the risk of cardiac arrhythmias and death. It is caused by decreased renal function or the use of renin–angiotensin–aldosterone inhibitors in patients with CKD and T2DM. Follow-up studies from several clinical trials and meta-analyses have found that SGLT2is may reduce the incidence of severe hyperkalemia. The mechanisms are considered to involve potassium co-excretion, increasing aldosterone level, and the indirect effects of preserved kidney function [17]. Considering that only a few real-world studies on hyperkalemia have been conducted, our study provides additional evidence that SGLT2is reduce the risk of hyperkalemia without causing hypokalemia.

In this study, the risk of diabetic ketoacidosis was evaluated. Even though the incidence of diabetic ketoacidosis was higher in the SGLT2i group than in the DPP4i group, the difference was not statistically significant. Euglycemic diabetic ketoacidosis is primarily caused by changes in insulin/glucagon ratio, increased ketogenesis, glycosuria, and the ketone reabsorption effect of SGLT2i [18]. In our study, which included patients prescribed insulin, factors during the follow-up period, such as discontinuation of insulin, may have influenced the development of diabetic ketoacidosis. In clinical trials, SGLT2is have also been associated with an increased risk of hypovolemia [19], but this was not statistically significant in our study. Similarly, several real-world studies have not observed a significant relationship between SGLT2is and severe hypovolemia [20]. Mechanistically, SGLT2is are considered safe with respect to hypoglycemia when used as monotherapy, and our study showed consistent findings [21].

It has been hypothesized that SGLT2is may affect bone metabolism and increase the risk of fractures [22]. However, our study observed no significant increase in the risk of bone fracture. This might be more pronounced with canagliflozin, which is known to affect bone resorption [23]; however, it was not included in our study as it was voluntarily withdrawn from the market in South Korea. Notably, a recent meta-analysis also found that SGLT2is are not significantly associated with an increased risk of fractures [23], and our results further support these findings.

Our cardiovascular results are consistent with those of previous studies on several cardiovascular diseases. Similarly to our findings, studies based on the US healthcare database and multi-database cohort study [24] have reported a lower risk of MACE in patients treated with SGLT2is. Furthermore, the results of a meta-analysis of retrospective cohort studies consistently showed a lower risk of HHF [25]. This is particularly important as cardiovascular disease is the leading cause of death and complications in patients with diabetes [26]. In Korea, SGLT2 inhibitors have recently been approved as standard therapy for heart failure, further highlighting the importance of our findings. The consistency between our study and these previous findings supports the cardiovascular benefits of SGLT2is. 

Our study has the following limitations. Due to the nature of the claims data, laboratory values were not available, which may affect the reliability of baseline characteristics and renal outcome assessments. Even though we defined variables using codes from multiple domains and a score variable, e.g., the diabetes comorbidity severity index, to heighten the sensitivity, and applied a large-scale propensity score model using all available measured covariates, the unmeasured biases cannot be entirely ruled out [27]. To further strengthen the robustness of our findings, future research should consider incorporating approaches such as leveraging external datasets with laboratory and clinical measurements or applying causal inference framework, such as target trial emulation. 

Additionally, our study may be subject to misclassification due to the structured nature of code-based analyses. For example, in the case of bone fractures, our data source did not allow us to distinguish between traumatic and non-traumatic fractures. As the HIRA-CDM undergoes continuous refinement with more granular mapping, misclassification is expected to decrease, strengthening future research. However, further research is needed to validate the diagnostic codes used for these outcomes and to develop more effective algorithms for accurately identifying renal disease progression in real-world data.

Despite these limitations, our study provides additional evidence on the renal protective effectiveness of SGLT2is, utilizing a large sample from the Asian population and comprehensive study outcome measures. While the existing literature observed only a limited variable outcome for renal disease (mainly ESRD) [5,14], our study assessed a broader spectrum of renal outcomes, including AKI, CKD, dialysis, and kidney transplantation. However, further research is warranted to evaluate whether the renal benefits of SGLT2is vary according to diabetes severity, including stratification by baseline insulin use. Such subgroup analyses would help clarify the extent to which the observed protective effects are influenced by underlying disease severity. We believe that our findings offer in-depth insights into the benefits of SGLT2is and provide supporting evidence for their pleiotropic effects [28], which have been increasingly highlighted in recent studies. In the Korean clinical setting, where SGLT2 inhibitors have recently been approved as standard therapy for heart failure and chronic kidney disease, our study contributes additional evidence for these expanded indications and helps reinforce their role in national treatment guidelines.

## 5. Conclusions

Using the large-scale nationwide data from South Korea, we confirmed that SGLT2is are associated with a reduced risk of both the composite renal outcome and the individual outcomes of AKI and CKD compared to DPP4i. In addition, a consistent higher incidence of genital infection and a lower incidence of hyperkalemia were also observed among SGLT2i users. Our study reinforce previous findings and supports current clinical guidelines on the use of SGL2 inhibitors in Asian populations.

## Figures and Tables

**Table 1:** Comparison of baseline characteristics, comorbidity, and concomitant medications between the SGLT2 and DPP4 inhibitor groups before and after the propensity score matching.

Characteristics	Before PS Matching			After PS Matching		
	SGLT2i (n = 16,736)	DPP4i (n = 67,463)	SMD	SGLT2i (n = 13,649)	DPP4i (n = 35,043)	SMD
Female, n (%)	6544 (39.1)	29,279 (43.4)	−0.09	5473 (40.1)	14,087 (40.2)	0.00
Age group, n (%)						
<40	1523 (9.1)	2361 (3.5)	0.19	915 (6.7)	2348 (6.7)	0.04
40–59	8853 (52.9)	25,906 (38.4)	0.12	6920 (50.7)	17,802 (50.8)	0.01
60–74	5372 (32.1)	28,065 (41.6)	0.09	4886 (35.8)	12,370 (35.3)	0.01
≥75	988 (5.9)	11,131 (16.5)	−0.17	928 (6.8)	2523 (7.2)	−0.03
Medical history, n (%)						
Hyperlipidemia	14,410 (86.1)	56,265 (83.4)	0.07	11,738 (86.0)	30,242 (86.3)	−0.01
Hypertensive disorder	10,996 (65.7)	42,975 (63.7)	0.04	8885 (65.1)	22,848 (65.2)	0.00
Cerebrovascular disease	1004 (6.0)	5060 (7.5)	−0.06	846 (6.2)	2208 (6.3)	0.00
Heart disease	4720 (28.2)	13,763 (20.4)	0.18	3535 (25.9)	9251 (26.4)	−0.01
Atrial fibrillation	435 (2.6)	1214 (1.8)	0.05	328 (2.4)	841 (2.4)	0.00
Heart failure	1807 (10.8)	4588 (6.8)	0.14	1283 (9.4)	3364 (9.6)	−0.01
Ischemic heart disease	3029 (18.1)	8096 (12.0)	0.17	2238 (16.4)	5852 (16.7)	−0.01
Peripheral vascular disease	2544 (15.2)	13,155 (19.5)	−0.11	2211 (16.2)	5817 (16.6)	−0.01
Osteoporosis	1439 (8.6)	9512 (14.1)	−0.18	1297 (9.5)	3434 (9.8)	−0.01
Medication use, n (%)						
ACE inhibitor/ARB	6661 (39.8)	23,477 (34.8)	0.10	5255 (38.5)	13,386 (38.2)	0.00
Antithrombotic agents	7950 (47.5)	32,113 (47.6)	0.00	6442 (47.2)	16,540 (47.2)	0.00
Calcium channel blockers	5322 (31.8)	21,319 (31.6)	0.00	4313 (31.6)	11,039 (31.5)	0.00
Diuretics	3381 (20.2)	13,358 (19.8)	0.01	2689 (19.7)	7044 (20.1)	−0.01
Insulins	1205 (7.2)	5195 (7.7)	−0.02	983 (7.2)	2523 (7.2)	0.00
Lipid-modifying agents	8853 (52.9)	32,653 (48.4)	0.09	7111 (52.1)	18,292 (52.2)	0.00

Abbreviations: PS, propensity score; SGLT2i, sodium–glucose cotransporter-2 inhibitor group; DPP4i, dipeptidyl peptidase-4 inhibitor group; SMD, standardized mean difference; ACE, angiotensin-converting enzyme; ARB, angiotensin receptor blocker.

**Table 2:** Incidence rate and hazard ratio of primary and secondary outcomes for the SGLT2 and DPP4 inhibitor groups.

Outcomes	SGLT2i (n = 13,649)		DPP4i (n = 35,043)		HR [95% CI]
	Events, n	IR	Events, n	IR	
**Renal outcomes**					
Any kidney outcomes	950	54.75	2722	61.08	0.88 [0.81–0.96] *
Acute kidney injury	72	3.21	320	5.44	0.61 [0.46–0.81] *
Chronic kidney disease	134	6.07	511	8.87	0.74 [0.60–0.91] *
Dialysis	25	1.11	105	1.77	0.64 [0.39–1.01]
Kidney failure	17	0.75	78	1.31	0.63 [0.35–1.09]
Kidney transplantation	0	0.00	<5	<0.08	0.20 [NA–2.06]
**Safety outcomes**					
Urinary tract infection	901	49.41	2408	50.70	0.97 [0.89–1.06]
Genital infection	686	837	34.44	15.46	2.38 [2.12–2.68] *
Diabetic ketoacidosis	21	0.93	35	0.59	1.27 [0.68–2.30]
Hyperkalemia	58	2.58	306	5.19	0.49 [0.36–0.67] *
Hypokalemia	73	265	3.25	4.49	0.82 [0.61–1.09]
Hypovolemia	214	627	9.83	11.01	0.92 [0.77–1.09]
Hypoglycemia	46	134	2.04	2.26	0.97 [0.65–1.42]
Bone fracture	610	1714	30.54	32.95	0.91 [0.82–1.02]

Abbreviations: SGLT2i, sodium–glucose cotransporter-2 inhibitor group; DPP4i, dipeptidyl peptidase-4 inhibitor group; n, number; IR, incidence rate per 1000 person-year; HR, hazard ratio; CI, confidence interval. * Statistically significant.

**Table 3:** Incidence rate and hazard ratio of primary outcomes in subgroups with the cardiovascular and renal risk between the SGLT2 and DPP4 inhibitor users.

Renal Outcomes	SGLT2i with CV Risk (n = 12,980)	DPP4i with CV Risk (n = 33,362)	HR [95% CI]	SGLT2i with Renal Risk (n = 2678)	DPP4i with Renal Risk (n = 7202)	HR [95% CI]
	Event, (IR)	Event, (IR)		Event, (IR)	Event, (IR)	
Any kidney outcomes	904 (55.48)	2602 (62.36)	0.90 [0.83–0.98] *	–	–	–
Acute kidney injury	66 (3.10)	309 (5.54)	0.53 [0.39–0.72] *	15 (3.83)	104 (9.67)	0.40 [0.21–0.70] *
Chronic kidney disease	139 (6.65)	499 (9.15)	0.81 [0.66–0.998] *	37 (10.29)	177 (18.2)	0.68 [0.45–0.98] *
Dialysis	25 (1.17)	98 (1.74)	0.72 [0.44–1.14]	7 (1.72)	44 (3.91)	0.46 [0.17–1.05]
Kidney failure	17 (0.79)	65 (1.15)	0.75 [0.40–1.32]	0 (0.00)	<5 (<0.44)	0.17 [NA–2.70]
Kidney transplantation	0 (0.00)	<5 (<0.09)	0.25 [NA–5.13]	0 (0.00)	<5 (<0.44)	0.17 [NA–2.70]

Abbreviations: SGLT2i, sodium–glucose cotransporter-2 inhibitor group; DPP4i, dipeptidyl peptidase-4 inhibitor group; IR, incidence rate per 1000 person-year; HR, hazard ratio; CI, confidence interval; CV, cardiovascular; * Statistically significant.

## Data Availability

Data for the results available on reasonable request to the corresponding author. Inquires for original datasets should be directed to the Big Data Department of Health Insurance Review and Assessment Service (https://opendata.hira.or.kr).

## Supplementary Materials

The following supporting information can be downloaded at: https://www.mdpi.com/article/10.3390/jcm14103349/s1, Figure S1: Flowchart for the SGLT2i and DPP4i groups; Figure S2: Cohort definition scheme for the SGLT2i and DPP4i groups. The cohort definition scheme is identical for both the SGLT2i and DPP4i groups, except for the specific drug initiated at cohort entry (SGLT2i or DPP4i, respectively). The scheme consists of two assessment windows and a follow-up window (exclusion assessment window: assesses prior medication use to exclude patients who were previously prescribed any other antidiabetic drugs † before cohort entry. The exclusion assessment period was restricted to the period for the data that were available in the HIRA-CDM database. Covariate assessment window: evaluates baseline characteristics in the 365 days before cohort entry. Follow-up window: begins on Day +1 after cohort entry and continues until the occurrence of one of the censoring events). † Other antidiabetic drugs: any antidiabetic drugs except metformin, insulin, SGLT2 inhibitors, and DPP4 inhibitors. SGLT2i: sodium–glucose cotransporter 2 inhibitor; DPP4i: dipeptidyl peptidase-4 inhibitor. * The patients were censored based on the following events: (1) encountered any of the clinical outcomes, (2) prescribed other antidiabetic drugs as defined in the exclusion criteria, and (3) discontinued treatment; Figure S3: Scatter plots between before and after the propensity score adjustment between the SGLT2i and DPP4i groups. This figure illustrates how well PS matching is adjusted for confounding bias by balancing covariates between cohorts. The *x*-axis and *y*-axis represent standardized mean differences in propensity score matching before and after PS matching, respectively. The data points under 0.1 based on the *y*-axis of the plot indicate that PS matching significantly reduced covariate imbalances, demonstrating its effectiveness in minimizing bias; Figure S4: Scatter plots between before and after the propensity score adjustment between SGLT2i and DPP4i groups. This figure illustrates how well PS matching is adjusted for confounding bias by balancing covariates between cohorts. The *x*-axis and *y*-axis represent standardized mean differences in propensity score matching before and after PS matching, respectively. The data points under 0.1 based on the *y*-axis of the plot indicate that PS matching significantly reduced covariate imbalances, demonstrating its effectiveness in minimizing bias; Figure S5: Scatter plots between before and after the propensity score adjustment between the SGLT2i and DPP4i groups. This figure illustrates how well PS matching is adjusted for confounding bias by balancing covariates between cohorts. The *x*-axis and *y*-axis represent standardized mean differences in propensity score matching before and after PS matching, respectively. The data points under 0.1 based on the *y*-axis of the plot indicate that PS matching significantly reduced covariate imbalances, demonstrating its effectiveness in minimizing bias; Table S1: Code list of study variables used in definition; Table S2: Incidence rate and hazard ratio of cardiovascular outcomes for the cohort groups; Table S3: Hazard ratios of primary outcomes from sensitivity analyses between the SGLT2i and DPP4i groups; Table S4: Hazard ratios of secondary outcomes from sensitivity analyses between the SGLT2 inhibitor and DPP4 inhibitor-only groups; Table S5: Comparison of baseline characteristics, comorbidity profiles, and concomitant drugs of the SGLT2 and DPP4 groups with cardiovascular risk before/after the PS matching; Table S6: Incidence rate and hazard ratio of safety outcomes in subgroups with the cardiovascular risk between the SGLT2 and DPP4 inhibitor users; Table S7: Hazard ratios of primary outcomes from sensitivity analyses in subgroups with cardiovascular risk between the SGLT2 and DPP4 inhibitor users; Table S8: Hazard ratios of secondary outcomes from sensitivity analyses in subgroups with cardiovascular risk between the SGLT2 and DPP4 inhibitor users; Table S9: Comparison of baseline characteristics, comorbidity profiles, and concomitant drugs of the SGLT2 and DPP4 groups with renal risk before/after the PS matching; Table S10: Incidence rate and hazard ratio of safety outcomes in subgroups with the renal risk between the SGLT2 and DPP4 inhibitor users; Table S11: Hazard ratios of primary outcomes from sensitivity analyses in subgroups with renal risk between the SGLT2 and DPP4 inhibitor users; Table S12: Hazard ratios of secondary outcomes from sensitivity analyses in subgroups with renal risk between the SGLT2 and DPP4 inhibitor users.

## Abbreviations

The following abbreviations are used in this manuscript:

SGLT2i	Sodium–glucose co-transporter 2 inhibitors
DPP4i	Dipeptidyl peptidase-4 inhibitors
T2DM	Type 2 diabetes mellitus
AKI	Acute kidney injury
CKD	Chronic kidney disease
ESRD	End-stage renal disease
CRRT	Continuous renal replacement therapy
AMI	Acute myocardial infarction
HHF	Hospitalization with heart failure
PS	Propensity score
PY	Person-year
HR	Hazard ratio
CI	Confidence interval
