# Supplementary material for: Renal and Safety Outcomes of SGLT2 Inhibitors in Patients with Type 2 Diabetes: A Nationwide Observational Cohort Study
Source: J Clin Med. 2025 May 12;14(10):3349. doi: 10.3390/jcm14103349 (PMC12112461; doi:10.3390/jcm14103349)
Supplement: Supplementary file 1 [file jcm-14-03349-s001.zip › jcm-3573416-supplementary.pdf]

Supplemental file

Supplemental file to J Chang, C Kim et al. "Renal and Safety Outcomes of SGLT2 Inhibitors in Patients with Type 2 Diabetes: A Nationwide Observational Cohort Study"

Table S1. Code list of study variables used in definition

Variables	OMOP concept ID	Desc	Excl
Type 2 diabetes mellitus	201826 (Type 2 diabetes mellitus)	X	
	4193704 (Type 2 diabetes mellitus without complication)	X	
SGLT2 inhibitor	42962859 (ipragliflozin Oral Tablet)	X	
	43009020 (ipragliflozin)		
	1123890 (dapagliflozin; oral)	X	
	1123740 (empagliflozin; oral)	X	
	715887 (ertugliflozin; oral)	X	
Combination drug with SGLT2 and metformin	793297 (ertugliflozin / metformin Oral Product)	X	
	793299 (ertugliflozin / metformin Oral Tablet)	X	
	36248374 (empagliflozin / metformin Oral Product)	X	
	46287679 (empagliflozin / metformin Oral Tablet)	X	
	45775454 (dapagliflozin / metformin Extended Release Oral Tablet)	X	
	36248557 (dapagliflozin / metformin Oral Product)	X	
	43534751 (metformin and dapagliflozin)	X	
	1588671 (metformin and empagliflozin)	X	
	715784 (metformin and ertugliflozin)	X	
DPP4 inhibitor	42961500 (gemigliptin 50 MG Oral Tablet)	X	
	40239218 (linagliptin 5 MG Oral Tablet)	X	
	40166041 (saxagliptin 5 MG Oral Tablet)	X	
	19125045 (sitagliptin 25 MG Oral Tablet)	X	
	19125049 (sitagliptin 50 MG Oral Tablet)	X	
	19125041 (sitagliptin 100 MG Oral Tablet)	X	
	19129179 (vildagliptin 50 MG Oral Tablet)	X	
	43013924 (alogliptin 25 MG Oral Tablet)	X	
	19122137 (vildagliptin)		
	1580747 (sitagliptin)		
	40166035 (saxagliptin)		
	40239216 (linagliptin)		
	43013884 (alogliptin)		
	42960653 (anagliptin 100 MG Oral Tablet)	X	
	42960599 (teneligliptin 20 MG Oral Tablet)	X	
	35198118 (trelagliptin succinates)	X	
	35197921 (omarigliptin)		
	42960653 (anagliptin 100 MG Oral Tablet)	X	
	43009089 (gemigliptin)		
	43009070 (teneligliptin)		
	43009051 (evogliptin)		
	43008991 (anagliptin)		
	715821 (gemigliptin and rosuvastatin)		
	21600786 (saxagliptin; oral)	X	
	21600784 (sitagliptin; oral)	X	
	21600787 (alogliptin; oral)	X	
	21600785 (vildagliptin; oral)	X	
	43534752 (gemigliptin; oral)	X	
	1502094 (evogliptin; oral)	X	
	40251691 (linagliptin; oral)	X	
Combination drug with DPP4 and metformin	43013885 (alogliptin / metformin Oral Tablet)	X	
	40139098 (metformin / sitagliptin Oral Tablet)	X	
	42961497 (gemigliptin / Metformin Extended Release Oral Tablet)	X	
	42960596 (Metformin / teneligliptin Extended Release Oral Tablet)	X	
	42961328 (evogliptin / Metformin Extended Release Oral Tablet)	X	
	42960651 (anagliptin / Metformin Oral Tablet)	X	
	40231387 (metformin / saxagliptin Extended Release Oral Tablet)	X	
	42705857 (linagliptin / metformin Oral Tablet)	X	
	36785176 (Linagliptin / Metformin Delayed Release Oral Tablet)	X	
	21140122 (Metformin / vildagliptin Oral Tablet)	X	
	21600772 (metformin and sitagliptin)		
	40251676 (metformin and saxagliptin)		
	43534749 (metformin and alogliptin)		
	1501888 (metformin and evogliptin)		
	45893491 (metformin and gemigliptin)		
	40251677 (metformin and linagliptin)		
	21600773 (metformin and vildagliptin)		
Metformin	19106521 (metformin 250 MG Oral Tablet)	X	
	40164897 (metformin hydrochloride 1000 MG Oral Tablet)	X	
	40164929 (metformin hydrochloride 500 MG Oral Tablet)	X	
	40164939 (metformin hydrochloride 750 MG Extended Release Oral Tablet)	X	
	40164946 (metformin hydrochloride 850 MG Oral Tablet)	X	
	40220371 (Modified 24 HR metformin hcl 1000 MG Extended Release Oral Tablet)	X	
	21600747 (metformin; oral)		
Antidiabetic drugs except metformin, SGLT2 inhibitor, combination drug with metformin and SGLT2 inhibitor, and insulin	42953698 (glimepiride 1 MG / Metformin 250 MG Oral Tablet)	X	
	42953740 (glimepiride 2 MG / Metformin 500 MG Oral Tablet)	X	
	42953818 (glimepiride 2 MG / Metformin 500 MG Extended Release Oral Tablet)	X	
	42953917 (glimepiride 1 MG / Metformin 500 MG Oral Tablet)	X	
	42961487 (gemigliptin 25 MG / Metformin 500 MG Extended Release Oral Tablet)	X	
	42961490 (gemigliptin 50 MG / Metformin 1000 MG Extended Release Oral Tablet)	X	
	42961494 (gemigliptin 50 MG / Metformin 500 MG Extended Release Oral Tablet)	X	

	36887702 (Metformin 850 MG / sitagliptin 50 MG Oral Tablet)	X	
	42960593 (Metformin 1000 MG / teneligliptin 20 MG Extended Release Oral Tablet)	X	
	21081251 (Metformin 850 MG / vildagliptin 50 MG Oral Tablet)	X	
	21169719 (Metformin 1000 MG / vildagliptin 50 MG Oral Tablet)	X	
	43267262 (Metformin 500 MG / vildagliptin 50 MG Oral Tablet)	X	
	42922767 (0.5 ML dulaglutide 1.5 MG/ML Pen Injector)	X	
	42922959 (0.5 ML dulaglutide 3 MG/ML Pen Injector)	X	
	42961500 (gemigliptin 50 MG Oral Tablet)	X	
	21133671 (Gliclazide 60 MG Extended Release Oral Tablet)	X	
	42960773 (lobeglitazone 0.415 MG Oral Tablet)	X	
	42961179 (voglibose 0.2 MG Oral Tablet)	X	
	42961189 (voglibose 0.3 MG Oral Tablet)	X	
	40165997 (glyburide 2.5 MG / metformin hydrochloride 500 MG Oral Tablet)	X	
	40166002 (glyburide 5 MG / metformin hydrochloride 500 MG Oral Tablet)	X	
	42708086 (linagliptin 2.5 MG / metformin hydrochloride 1000 MG Oral Tablet)	X	
	42708090 (linagliptin 2.5 MG / metformin hydrochloride 850 MG Oral Tablet)	X	
	42708088 (linagliptin 2.5 MG / metformin hydrochloride 500 MG Oral Tablet)	X	
	40164891 (metformin hcl 1000 MG / sitagliptin 50 MG Oral Tablet)	X	
	42708172 (metformin hcl 1000 MG / sitagliptin 50 MG Extended Release Oral Tablet)	X	
	40164922 (metformin hcl 500 MG / sitagliptin 50 MG Oral Tablet)	X	
	42708176 (metformin hcl 500 MG / sitagliptin 50 MG Extended Release Oral Tablet)	X	
	42708168 (metformin hcl 1000 MG / sitagliptin 100 MG Extended Release Oral Tablet)	X	
	19077682 (glyburide 5 MG Oral Tablet)	X	
	19059797 (gliclazide 80 MG Oral Tablet)	X	
	19101729 (gliclazide 30 MG Extended Release Oral Tablet)	X	
	1597761 (glimepiride 1 MG Oral Tablet)	X	
	1597772 (glimepiride 2 MG Oral Tablet)	X	
	1597758 (glimepiride 3 MG Oral Tablet)	X	
	1597773 (glimepiride 4 MG Oral Tablet)	X	
	40239218 (linagliptin 5 MG Oral Tablet)	X	
	19107110 (nateglinide 90 MG Oral Tablet)	X	
	1502829 (nateglinide 120 MG Oral Tablet)	X	
	1525221 (pioglitazone 15 MG Oral Tablet)	X	
	40166041 (saxagliptin 5 MG Oral Tablet)	X	
	19125045 (sitagliptin 25 MG Oral Tablet)	X	
	19125049 (sitagliptin 50 MG Oral Tablet)	X	
	19125041 (sitagliptin 100 MG Oral Tablet)	X	
	19129179 (vildagliptin 50 MG Oral Tablet)	X	
	43534749 (metformin and alogliptin)		
	21600782 (pioglitazone; orals)		
	21600783 (Dipeptidyl peptidase 4 (DPP-4) inhibitorss)	X	
	45893491 (metformin and gemigliptin)		
	21600770 (metformin and pioglitazone)		
	40251676 (metformin and saxagliptin)		
	21600776 (acarbose; orals)		
	21600774 (pioglitazone and alogliptins)	X	
	715821 (gemigliptin and rosuvastatin)		
	21600758 (gliclazide; orals)	X	
	21600761 (glimepiride; orals)	X	
	21600756 (glipizide; orals)	X	
	1501756 (insulin glargine and lixisenatide; systemic)		
	21600796 (mitiglinide; orals)	X	
	21600791 (nateglinide; orals)	X	
	21600790 (repaglinide; orals)	X	
	21600786 (saxagliptin; orals)	X	
	21600784 (sitagliptin; orals)	X	
	21600778 (voglibose; orals)	X	
	1123609 (lixisenatide; parenterals)	X	
	1123633 (exenatide; parenterals)	X	
	1123739 (albiglutide; parenterals)	X	
	21600750 (glibenclamide; orals)	X	
	21600765 (Combinations of oral blood glucose lowering drugs)		
	21600767 (metformin and sulfonylureas)		
Antidiabetic drugs except metformin, DPP4 inhibitor, combination drug with metformin and DPP4 inhibitor, and insulin	42953698 (glimepiride 1 MG / Metformin 250 MG Oral Tablets)	X	
	42953740 (glimepiride 2 MG / Metformin 500 MG Oral Tablets)	X	
	42953818 (glimepiride 2 MG / Metformin 500 MG Extended Release Oral Tablet)	X	
	42953917 (glimepiride 1 MG / Metformin 500 MG Oral Tablets)	X	
	42922767 (0.5 ML dulaglutide 1.5 MG/ML Pen Injectors)	X	
	42922959 (0.5 ML dulaglutide 3 MG/ML Pen Injectors)	X	
	21133671 (Gliclazide 60 MG Extended Release Oral Tablets)	X	
	42960773 (lobeglitazone 0.415 MG Oral Tablets)	X	
	42961179 (voglibose 0.2 MG Oral Tablets)	X	
	42961189 (voglibose 0.3 MG Oral Tablets)	X	
	35158145 (ipragliflozin / sitagliptin Oral Tablet)		
	35157372 (ipragliflozin / sitagliptin Oral Tablet [Sujanu])		
	40165997 (glyburide 2.5 MG / metformin hydrochloride 500 MG Oral Tablets)	X	
	40166002 (glyburide 5 MG / metformin hydrochloride 500 MG Oral Tablets)	X	
	44785831 (dapagliflozin 10 MG Oral Tablets)	X	
	45774754 (empagliflozin 10 MG Oral Tablets)	X	
	19077682 (glyburide 5 MG Oral Tablets)	X	
	19059797 (gliclazide 80 MG Oral Tablets)	X	
	19101729 (gliclazide 30 MG Extended Release Oral Tablets)	X	

	1597761 (glimepiride 1 MG Oral Tablets)	X	
	1597772 (glimepiride 2 MG Oral Tablets)	X	
	1597758 (glimepiride 3 MG Oral Tablets)	X	
	1597773 (glimepiride 4 MG Oral Tablets)	X	
	19107110 (nateglinide 90 MG Oral Tablets)	X	
	1502829 (nateglinide 120 MG Oral Tablets)	X	
	1525221 (pioglitazone 15 MG Oral Tablets)	X	
	43534751 (metformin and dapagliflozin)		
	1588671 (metformin and empagliflozin)		
	21600782 (pioglitazone; orals)		
	21600770 (metformin and pioglitazone)		
	21600776 (acarbose; orals)		
	1123740 (empagliflozin; orals)	X	
	715887 (ertugliflozin; orals)	X	
	21600758 (gliclazide; orals)	X	
	21600761 (glimepiride; orals)	X	
	21600756 (glipizide; orals)	X	
	1501756 (insulin glargine and lixisenatide; systemics)	X	
	715760 (ipragliflozins)	X	
	21600796 (mitiglinide; orals)	X	
	21600791 (nateglinide; orals)	X	
	21600782 (pioglitazone; orals)	X	
	21600790 (repaglinide; orals)	X	
	21600778 (voglibose; orals)	X	
	1123609 (lixisenatide; parenterals)	X	
	1123633 (exenatide; parenterals)	X	
	1123739 (albiglutide; parenterals)	X	
	21600750 (glibenclamide; orals)	X	
	21600765 (Combinations of oral blood glucose lowering drugs)		
	1123627 (Sodium-glucose co-transporter 2 (SGLT2) inhibitorss)	X	
	1501756 (insulin glargine and lixisenatide; systemic)		
	21600767 (metformin and sulfonylureas)		
Sudden cardiac death	4048809 (Brainstem deaths)	X	
	321042 (Cardiac arrests)	X	
	442289 (Death in less than 24 hours from onset of symptomss)	X	
	4317150 (Sudden cardiac deaths)	X	
	4132309 (Sudden deaths)	X	
	437894 (Ventricular fibrillation)	X	X
Acute myocardial infarction	4329847 (Myocardial infarctions)	X	
	314666 (Old myocardial infarction)	X	X
Intracranial bleed hemorrhagic stroke	376713 (Cerebral hemorrhage)		
	432923 (Subarachnoid hemorrhage)		
	43530727 (Spontaneous cerebral hemorrhage)		
	4148906 (Spontaneous subarachnoid hemorrhage)		
	439847 (Intracranial hemorrhages)	X	
Ischemic stroke	372924 (Cerebral artery occlusion)		
	375557 (Cerebral embolism)		
	443454 (Cerebral infarctions)	X	
	441874 (Cerebral thrombosis)		
	4111715 (Occlusion and stenosis of cerebral arteries, not resulting in cerebral infarction)		
Heart failure	315295 (Congestive rheumatic heart failure)	X	X
	316139 (Heart failures)	X	
General cardiovascular risk	316866 (Hypertensive disorders)	X	
	432867 (Hyperlipidemias)	X	
	433736 (Obesity)	X	
Kidney disease	4030518 (Renal impairments)	X	
	35624383 (Disorder of renal parenchymas)	X	
	193253 (Nephritis)	X	
	36716270 (Cyst of kidneys)	X	
	40483287 (Disorder of kidney and/or ureter)		
	37399735 (Nephrosis co-occurrent and due to systemic lupus erythematosus)		
	198124 (Kidney disease)		
	4309006 (Kidney transplant failure and rejection)		
	37399017 (Hemorrhagic fever with renal syndrome)		
	443731 (Renal disorder due to type 2 diabetes mellitus)		
	45757772 (Congenital renal cyst)	X	X
	4173195 (Congenital nephritis)	X	X
	4172011 (Congenital nephrotic syndrome)	X	X
	4149398 (Congenital renal failure)	X	X
	40481595 (Congenital hemolytic uremic syndrome)	X	X
	4244939 (Congenital absence of renal papilla)	X	X
	4126105 (Congenital Fanconi syndrome)	X	X
	4262564 (Congenital hypoplasia of renal papilla)	X	X
	37111021 (Congenital membranous nephropathy due to maternal anti-neutral endopeptidase alloimmunization)	X	X
	36717532 (Congenital nephrotic syndrome due to congenital infection)	X	X
	36717553 (Congenital nephrotic syndrome due to diffuse mesangial sclerosis)		
	4030510 (Congenital nephrotic syndrome with focal glomerulosclerosis)	X	X
	37116655 (Congenital nephrotic syndrome, interstitial lung disease, epidermolysis bullosa syndrome)		
	439694 (Hypertensive heart and renal disease with both (congestive) heart failure and renal failure)	X	X
	4161421 (Hereditary nephritis)		
	36717238 (Glomerular disorder due to non-neuropathic heredofamilial amyloidosis)	X	X
	35622779 (Hereditary clear cell renal cell carcinoma)		

	45757746 (Hereditary diffuse crescentic glomerulonephritis)<br>45757750 (Hereditary focal and segmental glomerular lesions)<br>45757751 (Hereditary minor glomerular abnormality)<br>4265337 (Hereditary nephrogenic diabetes insipidus)<br>4302592 (Hereditary tubulointerstitial disorder)<br>4163736 (Non-progressive hereditary glomerulonephritis)<br>4128203 (Acquired renal cyst with neoplastic change)<br>4126428 (Acquired renal cyst without neoplastic change)<br>36717534 (Chronic kidney disease following excision of renal neoplasm)<br>4059590 (Glomerular disorders in neoplastic diseases)<br>4059128 (Renal tubulo-interstitial disorders in neoplastic diseases)	X<br><br>X<br>X<br>X<br>X<br>X<br>X<br>X<br>X<br>X<br>X<br>X<br>X<br>X<br>X	X<br><br>X<br>X<br>X<br>X<br>X<br>X<br>X<br>X<br>X<br>X<br>X<br>X<br>X<br>X
Renal failure	197320 (Acute renal failure syndromes)<br>192359 (Renal failure syndromes)<br>198185 (Chronic renal failures)<br>443919 (Hypertensive renal failures)<br>443614 (Chronic kidney disease stage 1)<br>443601 (Chronic kidney disease stage 2)<br>443597 (Chronic kidney disease stage 3)<br>443612 (Chronic kidney disease stage 4)<br>443611 (Chronic kidney disease stage 5)	X<br>X<br>X<br>X	
Acute kidney injury	197320 (Acute renal failure syndromes)<br>761083 (Acute injury of kidneys)	X<br>X	
Chronic renal disease	46271022 (Chronic kidney diseases)	X	
End stage renal disease	443611 (Chronic kidney disease stage 5s)	X	
Hemodialysis	Procedure<br>4120120 (Hemodialysis)<br>4197217 (Arteriovenous anastomosis for renal dialysis)<br>4183419 (Arteriovenous shunt for renal dialysis by external cannulas)<br>4141148 (Repair of acquired arteriovenous fistulas)<br>42537496 (Insertion of hemodialysis catheters)<br>4160832 (Insertion of tunneled dialysis catheter using fluoroscopic guidance)<br>42539666 (Insertion of tunneled hemodialysis catheters)<br>42872870 (Insertion of nontunneled hemodialysis catheters)<br>46270107 (Insertion of dialysis catheter into femoral veins)<br>4051326 (Intermittent hemodialysis)<br><br>Device<br>42872871 (Hemodialysis catheters)<br>4030449 (Catheter guide wires)<br>45757875 (Hemodialysis catheter, implantable)	 X<br>X<br>X<br>X<br>X<br>X<br>X<br>X<br>X<br>X<br><br><br>X<br>X<br>X	
Peritoneal dialysis	4324124 (Peritoneal dialysiss)<br>46271816 (Management of peritoneal dialysis)	X<br>X	
CRRT	4051329 (Continuous venovenous hemodialysis)<br>42537496 (Insertion of hemodialysis catheter)<br>4051328 (Continuous arteriovenous hemodialysis)<br>4051329 (Continuous venovenous hemodialysis)<br>4051330 (Continuous venovenous hemofiltration)<br>4084444 (Vascular cannula insertion)<br>4049844 (Continuous arteriovenous hemofiltration)	 <br>X<br>X	
Dialysis	4019967 (Dependence on renal dialysis)	X	
Kidney transplantation	Procedure<br>4197300 (Donor renal transplantations)<br><br>Condition<br>42539502 (Transplanted kidney presents)	 X<br><br><br>X	
	42357561 (Renal Transplantations)	X	
Urinary tract infection	4311853 (Upper urinary tract infections)	X	
	36102977 (Urinary tract infections)<br>36110718 (Genitourinary tract infection)<br>37003664 (Genitourinary tract infections and inflammations NEC)<br>36110736 (Urinary tract infections)	X<br>X<br>X<br>X	 X<br>X
	42494780 (Urinary tract infection, site not specified)	X	
Genital infection	4193182 (Female genital infections)<br>4193988 (Genital infections)<br>4207302 (Male genital infections)	X<br>X<br>X	
	36110718 (Genitourinary tract infection)<br>37003664 (Genitourinary tract infections and inflammations NEC)	X<br>X	X<br>X
Diabetic ketoacidosis	443727 (Diabetic ketoacidosis)<br>443734 (Ketoacidosis due to type 2 diabetes mellitus)<br>439770 (Ketoacidosis due to type 1 diabetes mellitus)	X<br>X<br>X	 <br>X
	35506494 (Diabetic ketoacidosis)<br>35531687 (Type I diabetes mellitus with ketoacidosis, exclude)	X	 X

Hyperkalemia	434610 (Hyperkalemia)		
Hypokalemia	437833 (Hypokalemia)		
Hypoglycemia	24609 (Hypoglycemia)		
Hypovolemia	37311319 (Hypovolemia)		
Bone fracture	75053 (Fracture of bones)	X	

Abbreviations: Desc, including the descendants codes based on code hierarchy; Excl, excluding the codes from each definition.

Table S2. Incidence rate and hazard ratio of cardiovascular outcomes for the cohort groups

Outcomes	SGLT2i (n = 13,649)	DPP4i (n = 35,043)	HR [95% CI]
	Event, (IR)	Event, (IR)	
All-cause mortality	97 (4.27)	382 (6.4)	0.72 [0.54-0.93]*
MACE	312 (13.95)	1,042 (17.8)	0.79 [0.68-0.91]*
HHF	172 (7.64)	597 (10.12)	0.75 [0.62-0.90]*
Stroke	131 (5.81)	423 (7.14)	0.93 [0.74-1.15]
AMI	54 (2.38)	155 (2.6)	0.82 [0.57-1.16]

Abbreviations: SGLT2i, sodium glucose cotransporter-2 inhibitor group; DPP4i, dipeptidyl peptidase-4 inhibitor group; IR, incidence rate per 1,000 person-year; HR, hazard ratio; CI, confidence interval; MACE, major adverse cardiovascular events; HHF, hospitalization for heart failure; AMI, acute myocardial infarction.
*Statistically significant.

Table S3. Hazard ratios of primary outcomes from sensitivity analyses between the SGLT2i and DPP4i groups

Analysis description	Any kidney outcomes	Acute kidney injury	Chronic kidney disease	Dialysis	Kidney failure	Kidney transplantation
**Main setting (ITT 1:4 Matching)**	0.88 [0.81-0.96]*	0.61 [0.46-0.81]*	0.74 [0.60-0.91]*	0.64 [0.39-1.01]	0.63 [0.35-1.09]	0.20 [NA-2.06]
**Follow-up Strategy (As treated)**	0.86 [0.77-0.97]*	0.67 [0.44-1.01]	0.72 [0.54-0.96]*	0.75 [0.32-1.61]	0.29 [0.04-1.18]	NA
**PS adjustment (1:1 matched)**	0.89 [0.80-0.99]*	0.63 [0.45-0.87]*	0.74 [0.57-0.96]*	0.62 [0.35-1.06]	0.56 [0.28-1.06]	NA
**PS adjustment (Stratification)**	0.89 [0.83-0.96]*	0.59 [0.46-0.76]*	0.75 [0.62-0.89]*	0.60 [0.38-0.90]*	0.66 [0.38-1.07]	0.29 [NA-3.04]

Abbreviations: SGLT2i, sodium glucose cotransporter-2 inhibitor group; DPP4i, dipeptidyl peptidase-4 inhibitor group; ITT, intention-to-treat; PS, propensity score; AT, as-treated.
*Statistically significant

Table S4. Hazard ratios of secondary outcomes from sensitivity analyses between the SGLT2 inhibitor and DPP4 inhibitor -only groups

Analysis description	Urinary tract infection	Genital infection	Diabetic ketoacidosis	Hyperkalemia	Hypokalemia	Hypovolemia	Hypoglycemia	Bone fracture	All-cause mortality	MACE	HHF	Stroke	AMI
**Main setting (ITT 1:4 Matching)**	0.97 [0.89-1.06]	2.38 [2.12-2.68]*	1.27 [0.68-2.30]	0.49 [0.36-0.67]*	0.82 [0.61-1.09]	0.92 [0.77-1.09]	0.97 [0.65-1.42]	0.91 [0.82-1.02]	0.72 [0.54-0.93]*	0.79 [0.68-0.91]*	0.75 [0.62-0.90]*	0.93 [0.74-1.15]	0.82 [0.57-1.16]
**Follow-up Strategy (As treated)**	0.96 [0.85-1.08]	3.06 [2.63-3.57]*	1.48 [0.56-3.80]	0.37 [0.23-0.57]*	0.58 [0.36-0.89]*	1.02 [0.79-1.31]	1.11 [0.63-1.88]	0.86 [0.74-0.996]*	0.76 [0.47-1.20]	0.86 [0.71-1.04]	0.79 [0.61-1.001]	1.05 [0.78-1.40]	0.81 [0.48-1.32]
**PS adjustment (1:1 matched)**	0.99 [0.89-1.11]	2.22 [1.90-2.59]*	1.50 [0.68-3.45]	0.52 [0.36-0.74]*	0.80 [0.54-1.17]	0.89 [0.71-1.11]	1.10 [0.67-1.83]	0.94 [0.83-1.08]	0.77 [0.55-1.09]	0.78 [0.66-0.93]*	0.70 [0.56-0.88]*	0.85 [0.64-1.11]	0.93 [0.61-1.43]
**PS adjustment (Stratification)**	0.94 [0.87-1.01]	2.21 [2.01-2.44]*	1.74 [1.04-2.83]	0.48 [0.37-0.63]*	0.79 [0.61-1.01]	0.93 [0.80-1.08]	0.89 [0.63-1.24]	0.93 [0.85-1.02]	0.67 [0.54-0.83]*	0.81 [0.71-0.91]*	0.78 [0.67-0.91]*	0.87 [0.72-1.05]	0.85 [0.63-1.13]

Abbreviations: MACE, 4-point major adverse cardiovascular events; CRRT, continuous renal replacement therapy; HHF, hospitalization for heart failure; AMI, acute myocardial infarction; AT, as-treated; ITT, intention-to-treat.
*Statistically significant

Table S5. Comparison of baseline characteristics, comorbidity profiles, and concomitant drugs of the SGLT2 and DPP4 groups with cardiovascular risk before/after the PS matching

Characteristic	Before PS adjustment			After PS adjustment		
	SGLT2i (n = 15,919)	DPP4i (n = 63,295)	SMD	SGLT2i (n = 12,980)	DPP4i (n = 33,362)	SMD
Female, n (%)	6,240 (39.2)	27,976 (44.2)	-0.10	5,283 (40.7)	13,445 (40.3)	0.01
Age group, n (%)						
< 40	1,353 (8.5)	1,962 (3.1)	0.19	805 (6.2)	2,069 (6.2)	0.00
40-59	8,373 (52.6)	23,609 (37.3)	0.13	6,542 (50.4)	16,881 (50.6)	0.00
60-74	5,222 (32.8)	26,837 (42.4)	0.09	4,699 (36.2)	11,910 (35.7)	0.01
≥ 75	971 (6.1)	10,887 (17.2)	-0.15	934 (7.2)	2,502 (7.5)	-0.01
Medical history, n (%)						
Hypertensive disorder	10,984 (69.0)	42,977 (67.9)	0.02	8,878 (68.4)	22,720 (68.1)	0.00
Cerebrovascular disease	987 (6.2)	4,937 (7.8)	-0.06	857 (6.6)	2,202 (6.6)	0.00
Heart disease	4,680 (29.4)	13,608 (21.5)	0.18	3,518 (27.1)	9,175 (27.5)	-0.01
Atrial fibrillation	430 (2.7)	1,203 (1.9)	0.05	324 (2.5)	834 (2.5)	0.00
Heart failure	1,799 (11.3)	4,557 (7.2)	0.14	1,285 (9.9)	3,370 (10.1)	-0.01
Ischemic heart disease	3,009 (18.9)	8,038 (12.7)	0.17	2,220 (17.1)	5,838 (17.5)	-0.01
Peripheral vascular disease	2,515 (15.8)	12,849 (20.3)	-0.12	2,194 (16.9)	5,672 (17)	0.00
Osteoporosis	1,401 (8.8)	9,304 (14.7)	-0.18	1,285 (9.9)	3,303 (9.9)	0.00
Medication use, n (%)						
ACE inhibitor / ARB	6,654 (41.8)	23,482 (37.1)	0.10	5,244 (40.4)	13,412 (40.2)	0.00
Antithrombotic agents	7,721 (48.5)	30,825 (48.7)	0.00	6,256 (48.2)	16,047 (48.1)	0.00
Beta blocking agents	3,454 (21.7)	10,190 (16.1)	0.14	2,557 (19.7)	6,706 (20.1)	-0.01
Calcium channel blockers	5,317 (33.4)	21,267 (33.6)	0.00	4,322 (33.3)	11,009 (33)	0.01
Diuretics	3,343 (21.0)	13,165 (20.8)	0.00	2,687 (20.7)	6,906 (20.7)	0.00
Lipid modifying agents	8,771 (55.1)	32,470 (51.3)	0.08	7,048 (54.3)	18,049 (54.1)	0.00

Abbreviations: PS, propensity score; SGLT2i, sodium glucose cotransporter-2 inhibitor group; DPP4i, dipeptidyl peptidase-4 inhibitor group; SMD, standard mean difference; MACE, major adverse cardiovascular events; ACE, angiotensin-converting enzyme; ARB, angiotensin receptor blocker.

Table S6. Incidence rate and hazard ratio of safety outcomes in subgroups with the cardiovascular risk between the SGLT2 and DPP4 inhibitor users.

Outcomes	SGLT2i with CV risk (n = 12,980)	DPP4i with CV risk (n = 33,362)	HR [95% CI]
	Event, (IR)	Event, (IR)	
Urinary tract infection	866 (50.42)	2,286 (51.21)	1.05 [0.96-1.15]
Genital infection	645 (34.15)	805 (15.67)	2.34 [2.08-2.64]*
Diabetic ketoacidosis	19 (0.89)	28 (0.50)	1.61 [0.81-3.16]
Hyperkalemia	60 (2.81)	309 (5.52)	0.46 [0.33-0.63]*
Hypokalemia	74 (3.47)	236 (4.21)	0.78 [0.58-1.04]
Hypovolemia	197 (9.54)	581 (10.75)	0.89 [0.74-1.07]
Hypoglycemia	42 (1.97)	127 (2.26)	1.05 [0.75-1.45]
Bone fracture	580 (30.70)	1,693 (34.50)	0.90 [0.82-0.98]*
All-cause mortality	96 (4.46)	375 (6.61)	0.78 [0.59-1.01]
MACE	313 (14.77)	995 (17.89)	0.84 [0.73-0.97]*
HHF	182 (8.53)	600 (10.71)	0.76 [0.63-0.92]*
Stroke	127 (5.94)	389 (6.91)	0.96 [0.76-1.19]
AMI	55 (2.56)	146 (2.58)	0.86 [0.60-1.22]

Abbreviations: SGLT2i, sodium glucose cotransporter-2 inhibitor group; DPP4i, dipeptidyl peptidase-4 inhibitor group; IR, incidence rate per 1,000 person-years; HR, hazard ratio; CI, confidence interval; MACE, major adverse cardiovascular events; HHF, hospitalization for heart failure; AMI, acute myocardial infarction.
*Statistically significant

Table S7. Hazard ratios of primary outcomes from sensitivity analyses in subgroups with cardiovascular risk between the SGLT2 and DPP4 inhibitor users.

Analysis description	Any kidney outcomes	Acute kidney injury	Chronic kidney disease	Dialysis	Kidney failure	Kidney transplantation
**Main setting (ITT 1:4 Matching)**	0.90 [0.83-0.98]*	0.53 [0.39-0.72]*	0.81 [0.66-0.998]*	0.72 [0.44-1.14]	0.75 [0.40-1.32]	0.25 [NA-5.13]
**Follow-up Strategy (As treated)**	0.89 [0.79-1.004]	0.59 [0.38-0.87]*	0.78 [0.58-1.05]	0.71 [0.33-1.40]	0.26 [0.06-0.76]*	0.14 [NA-6.75]
**PS adjustment (1:1 matched)**	0.93 [0.83-1.03]	0.58 [0.40-0.82]*	0.84 [0.65-1.08]	0.63 [0.35-1.12]	0.59 [0.29-1.16]	0.14 [NA-6.75]
**PS adjustment (Stratification)**	0.90 [0.83-0.96]*	0.58 [0.45-0.75]*	0.75 [0.62-0.90]*	0.59 [0.38-0.90]*	0.66 [0.38-1.07]	0.28 [NA-2.98]

Abbreviations: SGLT2i, sodium glucose cotransporter-2 inhibitor group; DPP4i, dipeptidyl peptidase-4 inhibitor group; ITT, intention-to-treat; PS, propensity score; AT, as-treated.
*Statistically significant

Table S8. Hazard ratios of secondary outcomes from sensitivity analyses in subgroups with cardiovascular risk between the SGLT2 and DPP4 inhibitor users

Analysis description	Urinary tract infection	Genital infection	Diabetic ketoacidosis	Hyperkalemia	Hypokalemia	Hypovolemia	Hypoglycemia	Bone fracture	All-cause mortality	MACE	HHF	Stroke	AMI
**Main setting (ITT 1:4 Matching)**	1.05 [0.96-1.15]	2.34 [2.08-2.64]*	1.61 [0.81-3.16]	0.46 [0.33-0.63]*	0.78 [0.58-1.04]	0.89 [0.74-1.07]	1.05 [0.75-1.45]	0.90 [0.82-0.98]*	0.78 [0.59-1.01]	0.84 [0.73-0.97]*	0.76 [0.63-0.92]*	0.96 [0.76-1.19]	0.86 [0.60-1.22]
**Follow-up Strategy (As treated)**	1.02 [0.90-1.15]	2.96 [2.54-3.47]*	0.96 [0.35-2.49]	0.40 [0.24-0.63]*	0.63 [0.40-0.96]*	0.86 [0.67-1.10]	1.02 [0.69-1.51]	0.90 [0.81-1.002]	0.78 [0.47-1.26]	0.82 [0.67-0.99]*	0.73 [0.57-0.93]*	1.11 [0.82-1.48]	0.65 [0.37-1.10]
**PS adjustment (1:1 matched)**	1.07 [0.95-1.19]	2.33 [1.99-2.74]*	1.30 [0.57-3.05]	0.45 [0.31-0.64]*	0.65 [0.45-0.93]*	0.80 [0.64-1.005]	0.98 [0.73-1.29]	0.92 [0.86-0.99]*	0.77 [0.54-1.10]	0.78 [0.65-0.93]*	0.73 [0.58-0.92]*	0.90 [0.68-1.20]	0.81 [0.54-1.21]
**PS adjustment (Stratification)**	0.96 [0.89-1.03]	2.20 [1.99-2.44]*	1.75 [1.03-2.89]	0.47 [0.36-0.61]*	0.79 [0.61-1.02]	0.93 [0.80-1.09]	0.89 [0.58-1.33]	0.87 [0.78-0.97]*	0.68 [0.54-0.84]*	0.82 [0.73-0.93]*	0.79 [0.67-0.92]*	0.90 [0.74-1.08]	0.85 [0.63-1.13]

Abbreviations: MACE, 4-point major adverse cardiovascular events; CRRT, continuous renal replacement therapy; HHF, hospitalization for heart failure; AMI, acute myocardial infarction; AT, as-treated; ITT, intention-to-treat.
*Statistically significant

Table S9. Comparison of baseline characteristics, comorbidity profiles, and concomitant drugs of the SGLT2 and DPP4 groups with renal risk before/after the PS matching

Characteristic	Before PS adjustment			After PS adjustment		
	SGLT2i (n = 3,124)	DPP4i (n = 13,763)	SMD	SGLT2i (n = 2,678)	DPP4i (n = 7,202)	SMD
Female, n (%)	1,334 (42.7)	6,703 (48.7)	-0.12	1,181 (44.1)	3,118 (43.3)	0.02
Age group, n (%)						
< 40	209 (6.7)	316 (2.3)	0.16	153 (5.7)	324 (4.5)	0.04
40-59	1,431 (45.8)	4,143 (30.1)	0.14	1,178 (44.0)	3,090 (42.9)	0.01
60-74	1,193 (38.2)	6,207 (45.1)	0.07	1,074 (40.1)	2,974 (41.3)	0.01
≥ 75	291 (9.3)	3,097 (22.5)	-0.15	273 (10.2)	814 (11.3)	-0.02
Medical history, n (%)						
Hyperlipidemia	2,877 (92.1)	12,442 (90.4)	0.06	2,464 (92.0)	6,640 (92.2)	-0.01
Hypertensive disorder	2,206 (70.6)	9,675 (70.3)	0.01	1,880 (70.2)	5,085 (70.6)	-0.01
Cerebrovascular disease	266 (8.5)	1,390 (10.1)	-0.06	230 (8.6)	576 (8.0)	0.02
Heart disease	1,100 (35.2)	4,060 (29.5)	0.12	900 (33.6)	2,463 (34.2)	-0.01
Atrial fibrillation	116 (3.7)	385 (2.8)	0.05	96 (3.6)	230 (3.2)	0.02
Heart failure	440 (14.1)	1,555 (11.3)	0.08	351 (13.1)	951 (13.2)	0.00
Ischemic heart disease	715 (22.9)	2,436 (17.7)	0.13	576 (21.5)	1,599 (22.2)	-0.01
Peripheral vascular disease	669 (21.4)	3,606 (26.2)	-0.11	584 (21.8)	1,620 (22.5)	-0.02
Osteoporosis	415 (13.3)	2,766 (20.1)	-0.18	372 (13.9)	1,044 (14.5)	-0.02
Medication use, n (%)						
ACE inhibitor / ARB	1,368 (43.8)	5,395 (39.2)	0.10	1,146 (42.8)	3,082 (42.8)	0.00
Antithrombotic agents	1,624 (52.0)	7,432 (54.0)	-0.04	1,393 (52.0)	3,723 (51.7)	0.01
Beta blocking agents	750 (24.0)	2,808 (20.4)	0.09	616 (23.0)	1,685 (23.4)	-0.01
Diuretics	775 (24.8)	3,510 (25.5)	-0.02	659 (24.6)	1,750 (24.3)	0.01
Lipid modifying agents	1,796 (57.5)	7,349 (53.4)	0.08	1,508 (56.3)	4,184 (58.1)	-0.04

Abbreviations: PS, propensity score; SGLT2i, sodium glucose cotransporter-2 inhibitor group; DPP4i, dipeptidyl peptidase-4 inhibitor group; SMD, standard mean difference; MACE, major adverse cardiovascular events; ACE, angiotensin-converting enzyme; ARB, angiotensin receptor blocker.

Table S10. Incidence rate and hazard ratio of safety outcomes in subgroups with the renal risk between the SGLT2 and DPP4 inhibitor users.

Outcomes	SGLT2i with renal risk (n = 2,678)	DPP4i with renal risk (n = 7,202)	HR [95% CI]
	Event, (IR)	Event, (IR)	
Urinary tract infection	173 (59.08)	519 (64.58)	0.93 [0.76-1.13]
Genital infection	121 (34.3)	166 (16.38)	2.41 [1.84-3.15]*
Diabetic ketoacidosis	< 5 (< 1.22)	8 (0.7)	2.23 [0.25-19.46]
Hyperkalemia	15 (3.73)	139 (12.58)	0.26 [0.14-0.46]*
Hypokalemia	19 (4.71)	80 (7.18)	0.67 [0.38-1.13]
Hypovolemia	43 (11.13)	155 (14.53)	0.70 [0.46-1.03]
Hypoglycemia	13 (3.23)	47 (4.22)	0.73 [0.35-1.40]
Bone fracture	126 (36.2)	364 (37.98)	1.04 [0.82-1.31]
All-cause mortality	23 (5.6)	107 (9.37)	0.63 [0.35-1.07]
MACE	77 (19.10)	291 (26.22)	0.75 [0.56-0.98]*
HHF	51 (12.57)	187 (16.72)	0.70 [0.50-0.98]*
Stroke	28 (6.87)	117 (10.35)	0.79 [0.50-1.22]
AMI	8 (1.95)	32 (2.81)	0.80 [0.33-1.80]

Abbreviations: SGLT2i, sodium glucose cotransporter-2 inhibitor group; DPP4i, dipeptidyl peptidase-4 inhibitor group; IR, incidence rate per 1,000 person-years; HR, hazard ratio; CI, confidence interval; MACE, major adverse cardiovascular events; HHF, hospitalization for heart failure; AMI, acute myocardial infarction.
*Statistically significant

Table S11. Hazard ratios of primary outcomes from sensitivity analyses in subgroups with renal risk between the SGLT2 and DPP4 inhibitor users

Analysis description	Acute kidney injury	Chronic kidney disease	Dialysis	Kidney failure	Kidney transplantation
**Main setting (ITT 1:4 Matching)**	0.40 [0.21-0.70]*	0.68 [0.45-0.98]*	0.46 [0.17-1.05]	NA [NA-NA]	0.17 [NA-2.70]
**Follow-up Strategy (As treated)**	0.56 [0.25-1.13]	0.64 [0.36-1.07]	0.42 [0.10-1.31]	NA [NA-NA]	NA
**PS adjustment (1:1 matched)**	0.31 [0.15-0.61]*	0.89 [0.55-1.43]	0.43 [0.15-1.07]	NA [NA-NA]	0.14 [NA-6.75]
**PS adjustment (Stratification)**	0.39 [0.22-0.64]*	0.53 [0.37-0.74]*	0.34 [0.14-0.70]*	NA [NA-NA]	0.29 [NA-5.40]

Abbreviations: SGLT2i, sodium glucose cotransporter-2 inhibitor group; DPP4i, dipeptidyl peptidase-4 inhibitor group; ITT, intention-to-treat; PS, propensity score; AT, as-treated.
*Statistically significant

Table S12. Hazard ratios of secondary outcomes from sensitivity analyses in subgroups with renal risk between the SGLT2 and DPP4 inhibitor users

Analysis description	Urinary tract infection	Genital infection	Diabetic ketoacidosis	Hyperkalemia	Hypokalemia	Hypovolemia	Hypoglycemia	Bone fracture	All-cause mortality	MACE	HHF	Stroke	AMI
**Main setting (ITT 1:4 Matching)**	0.93 [0.76-1.13]	2.41 [1.84-3.15]*	2.23 [0.25-19.46]	0.26 [0.14-0.46]*	0.67 [0.38-1.13]	0.70 [0.46-1.03]	0.73 [0.35-1.40]	1.04 [0.82-1.31]	0.63 [0.35-1.07]	0.75 [0.56-0.98]*	0.70 [0.50-0.98]	0.79 [0.50-1.22]	0.80 [0.33-1.80]
**Follow-up Strategy (As treated)**	0.97 [0.76-1.24]	3.61 [2.58-5.10]*	0.29 [NA-7.20]	0.32 [0.14-0.65]*	0.37 [0.13-0.90]*	0.86 [0.49-1.46]	0.70 [0.26-1.65]	1.08 [0.79-1.47]	0.19 [0.05-0.56]*	0.89 [0.61-1.28]	0.76 [0.48-1.18]	1.75 [0.99-3.05]	1.13 [0.34-3.30]
**PS adjustment (1:1 matched)**	0.92 [0.72-1.18]	2.09 [1.48-2.99]*	NA	0.31 [0.15-0.58]*	0.48 [0.23-0.94]	0.76 [0.47-1.23]	0.82 [0.33-1.98]	0.97 [0.72-1.30]	0.62 [0.32-1.14]	0.85 [0.60-1.19]	0.80 [0.53-1.21]	0.85 [0.48-1.49]	0.89 [0.33-2.32]
**PS adjustment (Stratification)**	0.93 [0.79-1.10]	2.20 [1.75-2.75]*	0.75 [0.17-2.40]	0.26 [0.15-0.43]*	0.73 [0.44-1.16]	0.88 [0.63-1.21]	0.92 [0.50-1.62]	0.93 [0.76-1.13]	0.59 [0.37-0.89]*	0.74 [0.58-0.94]*	0.77 [0.57-1.03]	0.80 [0.53-1.17]	0.53 [0.22-1.09]

Abbreviations: MACE, 4-point major adverse cardiovascular events; CRRT, continuous renal replacement therapy; HHF, hospitalization for heart failure; AMI, acute myocardial infarction; AT, as-treated; ITT, intention-to-treat.
*Statistically significant

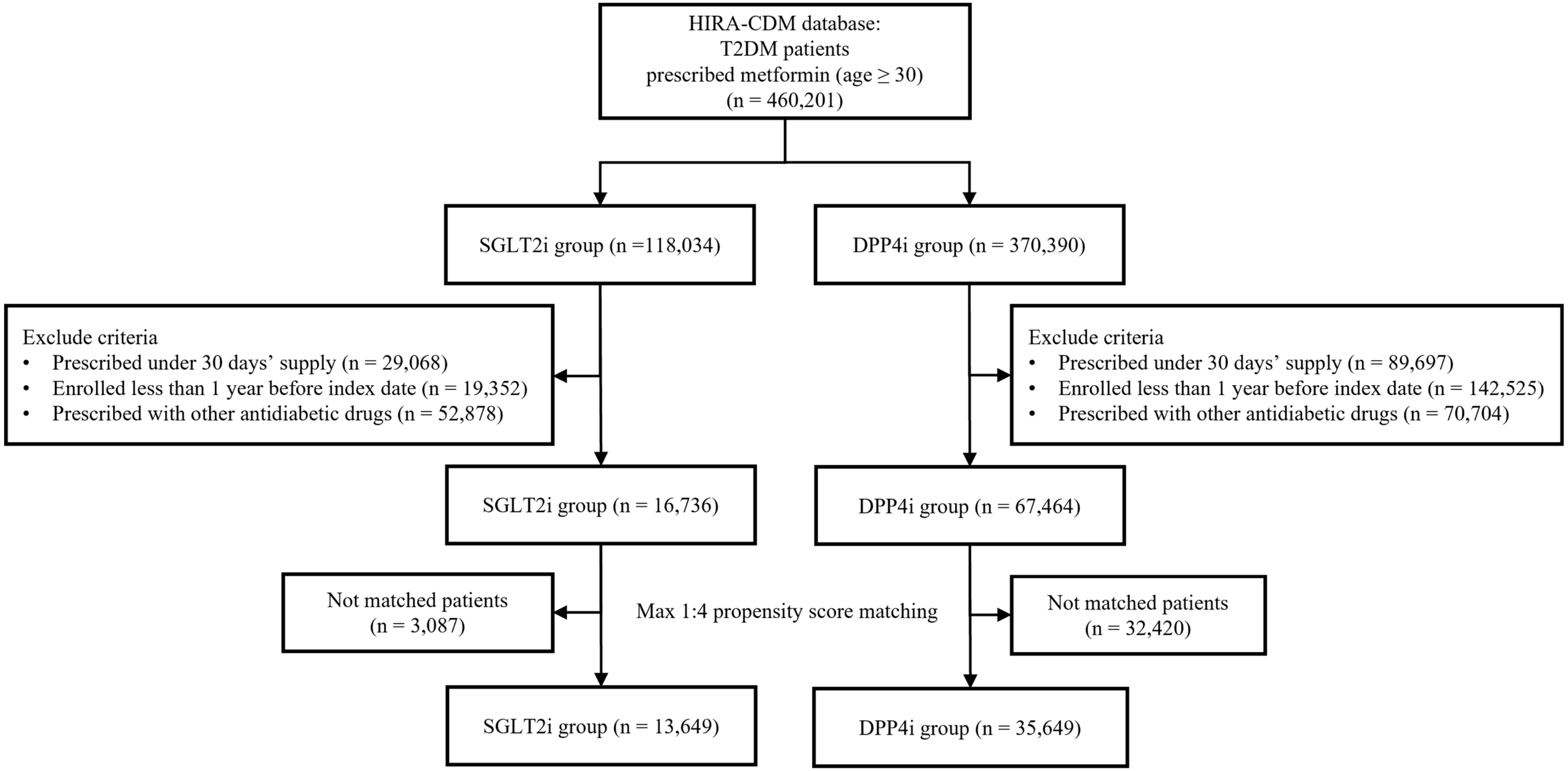

Figure S1 Flowchart for the SGLT2i and DPP4i groups

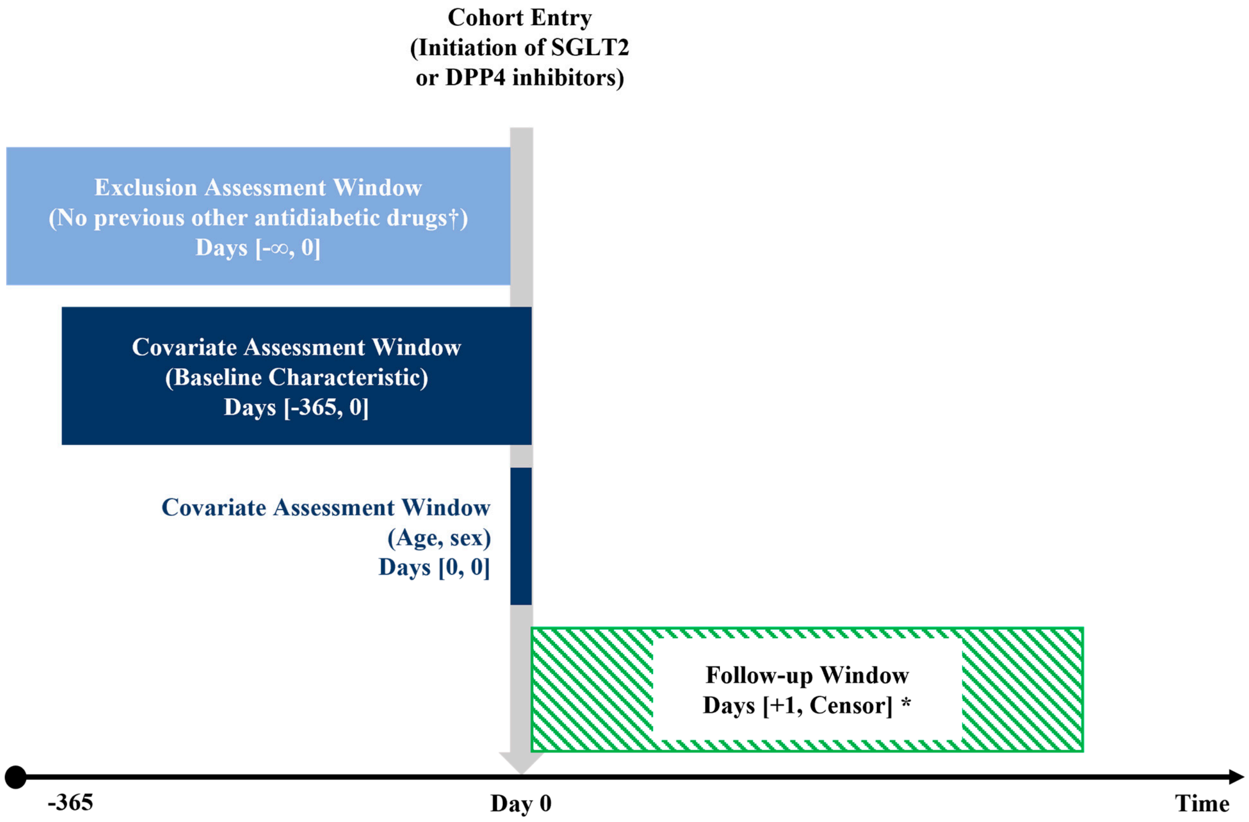

Figure S2 Cohort definition scheme for the SGLT2i and DPP4i groups. The cohort definition scheme is identical for both the SGLT2i and DPP4i groups, except for the specific drug initiated at cohort entry (SGLT2i or DPP4i, respectively). The scheme consists of two assessment windows and a follow-up window (Exclusion Assessment Window: Assesses prior medication use to exclude patients who were previously prescribed any other antidiabetic drugs† before cohort entry. The exclusion assessment period was restricted to the period for the data were available in the HIRA-CDM database. Covariate Assessment Window: Evaluates baseline characteristics in the 365 days before cohort entry. Follow-up Window: Begins on Day +1 after cohort entry and continues until the occurrence of one of the censoring events). †Other antidiabetic drugs: any antidiabetic drugs except metformin, insulin, SGLT2 inhibitors, and DPP4 inhibitors. SGLT2i: sodium-glucose cotransporter 2 inhibitor; DPP4i: dipeptidyl peptidase 4 inhibitor. *The patients were censored based on the following events: (1) encountered any of the clinical outcomes, (2) prescribed other antidiabetic drugs as defined in the exclusion criteria, and (3) discontinued treatment.

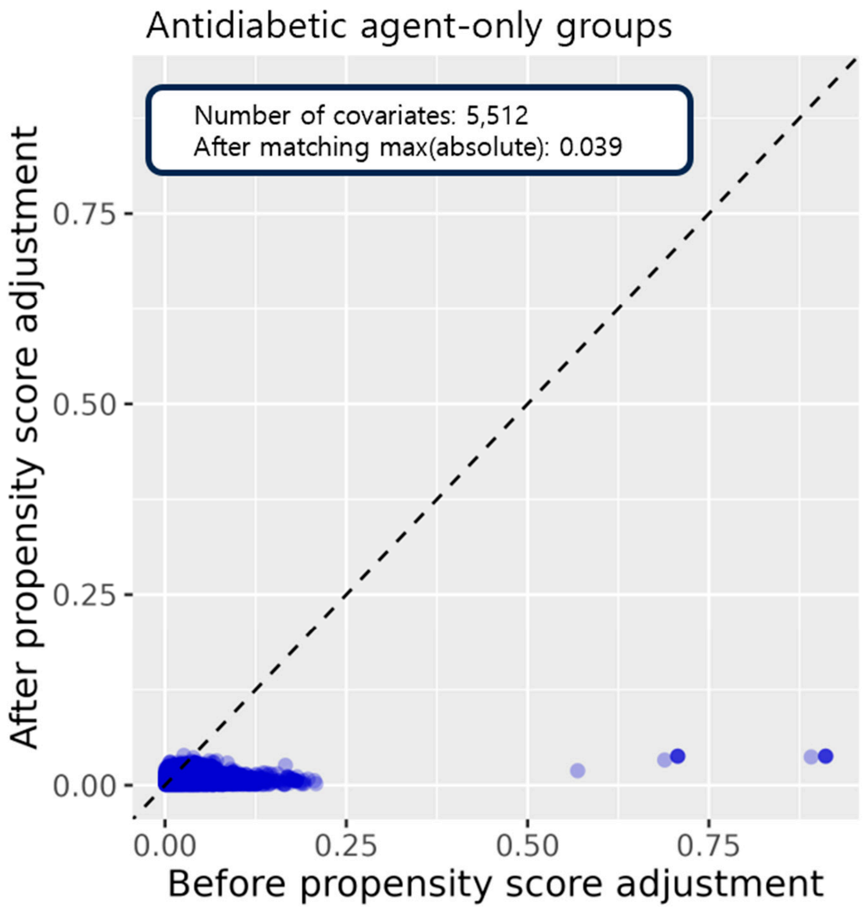

Figure S3. Scatter plots between before and after the propensity score adjustment between the SGLT2i and DPP4i groups. This figure illustrates how well PS matching adjusted for confounding bias by balancing covariates between cohorts. The x-axis and y-axis represent standardized mean differences of propensity score matching before and after PS matching, respectively. The data points under 0.1 based on the y-axis of the plot indicate that PS matching significantly reduced covariate imbalances, demonstrating its effectiveness in minimizing bias.

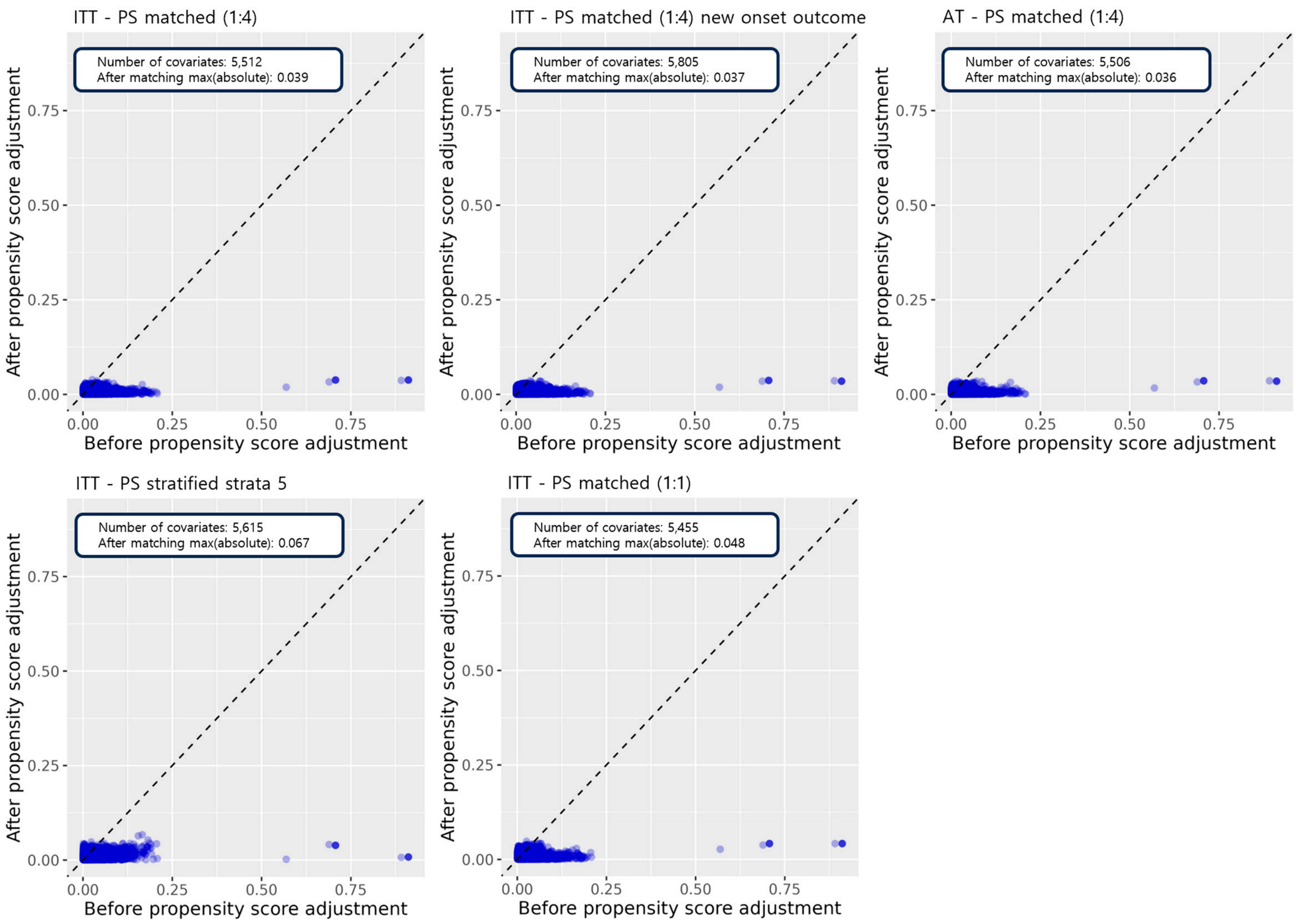

Figure S4. Scatter plots between before and after the propensity score adjustment between SGLT2i and DPP4i groups. This figure illustrates how well PS matching adjusted for confounding bias by balancing covariates between cohorts. The x-axis and y-axis represent standardized mean differences of propensity score matching before and after PS matching, respectively. The data points under 0.1 based on the y-axis of the plot indicate that PS matching significantly reduced covariate imbalances, demonstrating its effectiveness in minimizing bias.

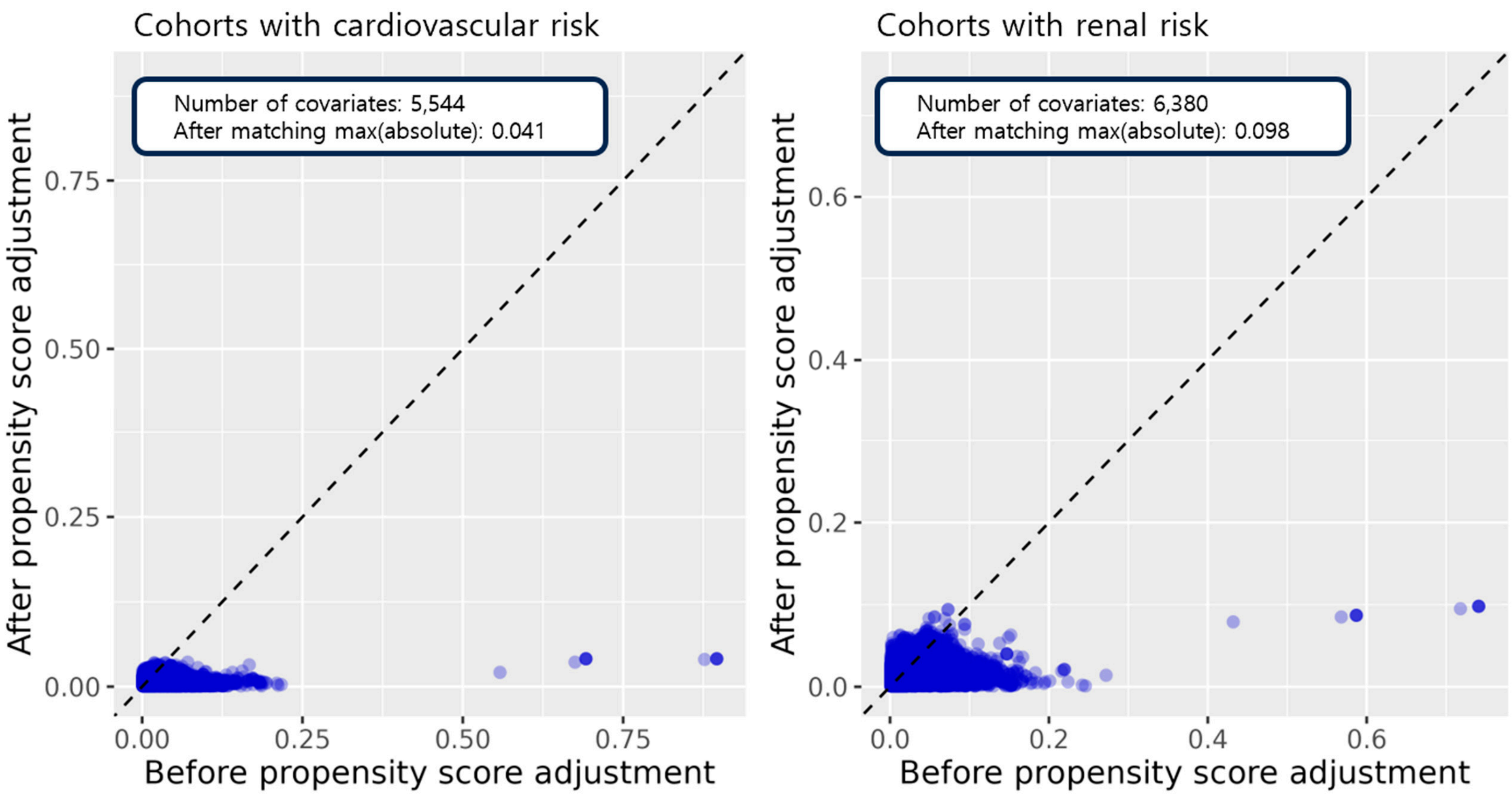

Figure S5. Scatter plots between before and after the propensity score adjustment between the SGLT2i and DPP4i groups. This figure illustrates how well PS matching adjusted for confounding bias by balancing covariates between cohorts. The x-axis and y-axis represent standardized mean differences of propensity score matching before and after PS matching, respectively. The data points under 0.1 based on the y-axis of the plot indicate that PS matching significantly reduced covariate imbalances, demonstrating its effectiveness in minimizing bias.
